# Effects of concomitant use of prasugrel with edoxaban on bleeding time, pharmacodynamics, and pharmacokinetics of edoxaban in healthy elderly Japanese male subjects: a clinical pharmacology study

**DOI:** 10.1186/s12959-020-00223-0

**Published:** 2020-06-12

**Authors:** Ippei Ikushima, Takaaki Akasaka, Yoshiyuki Morishima, Atsushi Takita, Tomoko Motohashi, Tetsuya Kimura

**Affiliations:** 1 Sumida Hospital, 29-1, Honjo 1-Chome, Sumida-ku, Tokyo, 130-0004 Japan; 2 Medical Science Department, Daiichi Sankyo Co., Ltd., 3-5-1 Nihonbashi Honcho, Chuo-ku, Tokyo, 103-8426 Japan; 3 Biostatistics & Data Management Department, Daiichi Sankyo Co., Ltd., 1-2-58 Hiromachi, Shinagawa-ku, Tokyo, 140-8710 Japan

**Keywords:** Edoxaban, Prasugrel, Healthy male subjects, Elderly Japanese, Bleeding time, Pharmacodynamics, Pharmacokinetics

## Abstract

**Background:**

Dual therapy with a direct oral anticoagulant (DOAC) plus a P2Y_12_ receptor inhibitor is recommended in patients with nonvalvular atrial fibrillation who undergo percutaneous coronary intervention. Thus, we evaluated the effects of DOAC edoxaban plus P2Y_12_ receptor inhibitor prasugrel on bleeding time (BT), and pharmacodynamics (PD) and pharmacokinetics (PK) of edoxaban in healthy elderly Japanese male subjects.

**Methods:**

A single-center, clinical pharmacology study with randomized, open-label, repeated dosing enrolled 24 participants in two groups of 12 receiving 30 mg edoxaban once daily for 3 days; then 30 mg edoxaban plus 2.5 mg prasugrel (Group 1) or 30 mg edoxaban plus 3.75 mg prasugrel (Group 2) once daily for 5 days. Primary endpoint was BT by the Ivy method. Secondary endpoints were the PD parameters of prothrombin time (PT), activated partial thromboplastin time (aPTT), prothrombin fragment F1 + 2 (F1 + 2), and P2Y_12_ reaction units (PRU), and PK profiles of edoxaban alone and in combination with prasugrel.

**Results:**

Geometric least squares mean of BT ratios (vs. baseline) for 3-day edoxaban treatment were 1.097 (90% confidence interval (CI) 0.911–1.321) in Group 1 and 1.327 (90% CI 1.035–1.703) in Group 2; for 5-day edoxaban plus 2.5 mg and 3.75 mg prasugrel, they were 1.581 (90% CI 1.197–2.087) and 1.996 (90% CI 1.482–2.690), respectively. Contributions of prasugrel to the effects (edoxaban + prasugrel/edoxaban) were 1.442 (90% CI 1.096–1.897) in Group 1 and 1.504 (90% CI 1.172–1.930) in Group 2. Edoxaban prolonged PT and aPTT and decreased F1 + 2. Adding on prasugrel did not appreciably change PT, aPTT, or F1 + 2. Prasugrel reduced PRU, whereas edoxaban had no effect on PRU. We recorded 26 adverse events; 23 were treatment-emergent (positive fecal occult blood test). All participants with adverse events recovered during follow-up.

**Conclusions:**

Coadministration of 2.5 mg and 3.75 mg prasugrel with 30 mg edoxaban prolonged BT in healthy elderly Japanese male subjects. The effect was dependent on the dose of prasugrel. Prasugrel did not affect PD or PK profiles of edoxaban. Edoxaban had no effect on PD of prasugrel.

**Trial registration:**

Japan Registry of Clinical Trials No. jRCTs071190006; registration date, 26-April-2019.

## Background

In patients with nonvalvular atrial fibrillation (NVAF), especially in those of advanced aged, coronary artery disease is common. In the All Nippon AF in the Elderly (ANAFIE) registry of 32,726 Japanese patients ≥75 years of age, 5600 (17.1%) had angina pectoris and 1874 (5.7%), a myocardial infarction [1].

The European Heart Rhythm Association, a branch of the European Society of Cardiology issued a 2018 joint European consensus document on the management of antithrombotic therapy in atrial fibrillation (AF) patients presenting with acute coronary syndrome and undergoing percutaneous coronary intervention (PCI) [2]. The document recommends as short a period as possible for triple therapy with an oral anticoagulant plus dual antiplatelet agents (a P2Y_12_ receptor inhibitor and aspirin), followed by dual therapy with an oral anticoagulant plus a single antiplatelet agent (P2Y_12_ receptor inhibitor).

The direct oral anticoagulant (DOAC) used in our study was edoxaban, a direct inhibitor of activated blood coagulation factor X, indicated to reduce the risk of stroke and systemic embolism in patients with NVAF [3, 4]. Edoxaban is available in a standard dose of 60 mg and a reduced dose of 30 mg for patients with low body weight (≤60 kg), moderate or severe renal impairment (creatinine clearance 15–50 mL/min), or concomitant use of potent P-glycoprotein inhibitors [4, 5]. According to baseline clinical characteristics of patients taking edoxaban enrolled in the ANAFIE registry (all 75 years of age or older), 82.2% of them were taking this reduced 30 mg dose [1].

The P2Y_12_ receptor inhibitor administered with edoxaban in this study was prasugrel, indicated for patients with acute coronary syndrome, stable angina, or old myocardial infarction who are to be managed with PCI in Japan [6, 7]. Compared to clopidogrel, prasugrel is a more potent P2Y_12_ receptor inhibitor [6–9]. In Japan, the doses of prasugrel (loading dose, 20 mg; standard daily maintenance dose, 3.75 mg; reduced daily maintenance dose, 2.5 mg for patients with low body weight ≤ 50 kg) are lower than those approved in western countries (loading dose, 60 mg; daily maintenance dose, 10 mg), and the bleeding risk of prasugrel was shown to be similar to that of clopidogrel [8, 9]. Recently, a retrospective analysis of an observational cohort study reported that in Japanese AF patients undergoing PCI, prasugrel as a part of triple therapy with aspirin and an oral anticoagulant had not increased the risk of bleeding compared to clopidogrel [10]. Besides this observational study, a phase 3b trial [11] was conducted to analyze the safety of edoxaban in combination with a P2Y_12_ receptor inhibitor in AF patients undergoing PCI. The study demonstrated that edoxaban plus a P2Y_12_ receptor inhibitor was non-inferior for bleeding events compared with a vitamin K antagonist in combination with a P2Y_12_ receptor inhibitor and aspirin without significant differences in ischemic events. However, data on the combination of edoxaban and prasugrel are very limited.

Here, we aimed to assess the effects of edoxaban plus prasugrel on bleeding time (BT), and pharmacodynamic (PD) and pharmacokinetic (PK) profiles of edoxaban in healthy elderly Japanese male subjects between the ages of 65 and 80.

## Methods

### Study population

The target sample size for the study population was 24 healthy elderly Japanese male subjects between the ages of 65 and 80 years, weighing 40 kg–60 kg (body mass index [BMI], 18.5 kg/m^2^–25.0 kg/m^2^). Subjects were excluded from participation in the study for any of the following reasons: presence of a bleeding disorder (history of intracranial bleeding, gastrointestinal bleeding, vitreous hemorrhage, retinal bleeding, hemoptysis, hematemesis, hematuria, bloody stools, hemorrhoids), prolonged BT of over 9.5 min, and prolonged prothrombin time (PT) and activated partial thromboplastin time (aPTT) exceeding the upper limit of normal values, a positive finding in fecal occult blood test or urine occult blood test, chronic kidney disease (estimated glomerular filtration rate (eGFR) of less than 60 mL/min/1.73 m^2^), or use or consumption any of the following within 7 days before the examination: inhibitors or inducers of the drug-metabolizing enzyme CYP3A, inhibitors of P-glycoprotein, and grapefruit or any food products containing the fruit.

### Study design

This study was a randomized, open-label, parallel-group, repeated-dose study conducted at a single center. Subjects who were eligible and agreed to participate in this study were admitted to the hospital for the length of the study and randomly assigned to two groups: Group 1, 30 mg edoxaban (dose for NVAF patients weighing 60 kg or less) plus 2.5 mg prasugrel (reduced maintenance dose in Japan); Group 2, 30 mg edoxaban plus 3.75 mg prasugrel (standard maintenance dose in Japan).

The study design, schedule of procedures, and blood sample collections are summarized in Table 1. This study was conducted at Sumida Hospital in Tokyo, Japan, from May to June 2019.

**Table 1:** Study design, schedule for BT, PD, and edoxaban PK evaluations

**Subjects**	Healthy elderly Japanese male subjects (Age: 65–80)	
**Study design**	Open-label, parallel-group, repeated-dose	
**Sample size**	24 Subjects randomized into two groups	
	Group 1, *n* = 12 30 mg Edoxaban + 2.5 mg Prasugrel	Group 2, *n* = 12 30 mg Edoxaban + 3.75 mg Prasugrel
**Treatments**	Stage 1: all participants Day 1, 2, 3: 30 mg Edoxaban once daily	
	Stage 2: Day 4, 5, 6, 7, 8 Group 1: 30 mg Edoxaban + 2.5 mg Prasugrel once daily	Stage 2: Day 4, 5, 6, 7, 8 Group 2: 30 mg Edoxaban + 3.75 mg Prasugrel once daily
**Schedule of procedures and blood sample collections**		
**Bleeding time (BT)**^**a**^**& PD Evaluation**^**b**^	Day 0: Day of admission Day 3: Three hours after administration of edoxaban Day 8: Three hours after administration of edoxaban + prasugrel	
**Edoxaban PK**	Day 1: Pre-administration of study drug Day 3: Pre-administration and 0.25, 0.5, 1, 2, 3, 4, 8, 12, 24 h after administration of edoxaban Day 8: Pre-administration and 0.25, 0.5, 1, 2, 3, 4, 8, 12, 24 h after administration of edoxaban + prasugrel	

^a^Ivy Method

^b^PD indices: prothrombin time (PT), activated partial thromboplastin time (aPTT), prothrombin fragment 1 + 2 (F1 + 2), and P2Y_12_ reaction units (PRU)

### Bleeding time

We measured BT by the Ivy method [12] using a needle type of BD Microtainer Contact-Activated Lancet device (Becton, Dickinson and Company, USA). The incision was made in the lateral aspect of the forearm by puncturing at one arbitrary site with the device. After the incision, we blotted the blood every 30 s with filter paper until the bleeding reached hemostasis and recorded the time from the puncture to the end of bleeding. When the time to hemostasis exceeded 10 min, it was recorded as such, ending the observation. The schedule of BT evaluations is included in Table 1. Baseline BT was measured on the day of admission at approximately the same time of day as the BT evaluations on Day 3 and Day 8, 3 hours after the administration of study drug(s).

### Pharmacodynamics

Effects of edoxaban alone and edoxaban plus prasugrel on the parameters of blood coagulation and platelet aggregation were assessed by determination of PT, aPTT, prothrombin fragment 1 + 2 (F1 + 2), and P2Y_12_ reaction units (PRU). Blood was collected in a vacuum blood collection tube containing 3.2% sodium citrate, and the schedule for the blood sample collection is shown in Table 1. Blood coagulation marker PT was measured by PT reagent Thrombocheck-PT in system CS-200i (Sysmex Corporation, Japan); aPTT by aPTT reagent HemosIL SynthASil in system ACL TOP Base (IL, Japan; Instrumentation Laboratory, USA) and F1 + 2 by ELISA Enzygnost F1 + 2 monoclonal reagent in system Behring ELISA Processor III and DiaSorin (BIT Analytical Instruments GmbH, Germany). These assays were carried out at LSI Medience Corporation (Tokyo, Japan). We measured PRU with VerifyNow system (Instrumentation Laboratory, Bedford, MA, USA) at Sumida Hospital.

### Pharmacokinetics

Blood for the determination of plasma edoxaban concentrations was collected from a forearm vein and deposited in a vacuum blood collection tube containing heparin sodium. Blood was collected before the administration and 0.25, 0.5, 1, 2, 3, 4, 8, 12, and 24 h after the drug administration on Day 1, Day 3, and Day 8 (Table 1). Plasma samples were obtained by centrifugation, and the samples were immediately frozen at − 20 °C or lower. Plasma edoxaban concentrations were measured by a liquid chromatography–tandem mass spectrometry system (SCIEX, Framingham, MA, USA) at Shin Nippon Biomedical Laboratories, Ltd. (Wakayama, Japan).

### Statistical analysis

The sample size selected was 24 (12 in each group) based on feasibility. According to results of phase 1 studies of edoxaban and prasugrel, inter-individual variations in BT and BT ratio before and after the administration were 20 to 45%, and intra-individual variations were estimated to be 20 to 30%. Under this estimation, effects of drugs on bleeding time could be analyzed with a certain degree of accuracy. All statistical analyses were performed using SAS Ver. 9.4 (SAS Institute Inc., Cary, NC, USA).

Summary statistics were calculated for baseline and postdose values for the primary endpoint of BT and secondary endpoints of PD indices (PT, aPTT, F1 + 2, PRU) for each target population. Furthermore, we calculated summary statistics of plasma edoxaban concentrations and estimated pharmacokinetic parameters (C_max,ss_, steady state maximum plasma concentration; AUC_tau,ss_, steady state area under the plasma concentration-time curve; t_max,ss_, steady state time to reach maximum plasma concentration) using a noncompartmental analysis engine of Phoenix WinNonlin Ver. 8.1 based on Model 1200 (Extravascular). AUC_tau,ss_ was calculated by the linear-Log trapezoidal method.

For the primary endpoint analysis, a logarithmically converted value of BT for each sample was the dependent variable in a linear mixed model with fixed and random effects. We calculated geometric least squares means (GLSM) of ratios of BT for the edoxaban alone and the edoxaban plus prasugrel combination, with 90% confidence intervals (CIs) for each treatment group.

For the secondary endpoints analysis, we used the logarithmically converted PK parameters (C_max,ss_, AUC_tau,ss,_ t_max,ss_) as dependent variables in a linear mixed model with fixed and variable effects. We calculated GLSM of these values for edoxaban administered alone and edoxaban plus prasugrel combination, with 90% CIs.

## Results

### Subject disposition and characteristics

A total of 24 healthy Japanese male subjects (age range 66–78 years) participated in this study: 12 administered 30 mg edoxaban plus 2.5 mg prasugrel (Group 1) and 12 administered 30 mg edoxaban plus 3.75 mg prasugrel (Group 2). All participants completed the study as planned. Demographic and clinical characteristics of participants are shown in Table 2.

**Table 2:** Baseline demographic and clinical characteristics

	All subjects	Group 1 30 mg edoxaban plus 2.5 mg prasugrel	Group 2 30 mg edoxaban plus 3.75 mg prasugrel
	*n* = 24	*n* = 12	*n* = 12
**Age (years)**			
Mean (SD)	72 (4)	71 (4)	73 (3)
65 to < 75, n (%)	16 (66.7)	9 (75.0)	7 (58.3)
75 to 80, n (%)	8 (33.3)	3 (25.0)	5 (41.7)
**Height (cm)**			
Mean (SD)	164.0 (5.7)	162.5 (5.7)	165.5 (5.6)
**Weight (kg)**			
Mean (SD)	56.4 (2.2)	55.8 (2.4)	57.0 (1.9)
> 50 to 60, n (%)	24 (100.0)	12 (100.0)	12 (100.0)
**BMI (kg/m**^**2**^**)**			
Mean (SD)	21.1 (1.6)	21.3 (1.6)	21.0 (1.6)
**eGFR (mL/min/1.73m**^**2**^**)**			
Mean (SD)	73.5 (7.0)	75.5 (6.6)	71.6 (7.1)
60 to < 90, n (%)	24 (100.0)	12 (100.0)	12 (100.0)
**CL**_**cr**_**(mL/min)**			
Mean (SD)	67.2 (7.2)	68.7 (6.1)	65.8 (8.1)
≥50, n (%)	24 (100.0)	12 (100.0)	12 (100.0)
**Medical history**^**a**^			
No, n (%)	10 (41.7)	5 (41.7)	5 (41.7)
Yes, n (%)	14 (58.3)	7 (58.3)	7 (58.3)

*SD* standard deviation, *IQR* interquartile range, *BMI* body mass index, *CL*_*cr*_ creatinine clearance

^a^Appendicitis, polyps in the colon, hemorrhoids, cataract, hepatitis A, tuberculosis, and inguinal hernia

All participant data was available for the final analyses.

### Bleeding time

Mean and median BTs (min) at baseline and after treatments are shown in Table 3.

**Table 3:** Mean and median bleeding times (min)

	Group 1 30 mg edoxaban plus 2.5 mg prasugrel	Group 2 30 mg edoxaban plus 3.75 mg prasugrel
	*n* = 12	*n* = 12
**Baseline**		
Mean (SD)	3.2 (0.8)	3.5 (1.1)
Median (IQR)	3.0 (2.5–3.8)	3.5 (2.5–4.5)
**Edoxaban administration**		
Mean (SD)	3.5 (0.6)	4.7 (1.6)
Median (IQR)	3.5 (3.0–4.0)	4.5 (3.3–6.0)
**Edoxaban plus prasugrel administration**		
Mean (SD)	5.4 (2.3)	7.3 (2.8)
Median (IQR)	5.0 (3.5–7.3)	7.5 (5.5–10.0)

*SD* standard deviation, *IQR* interquartile range

**Fig. 1:**
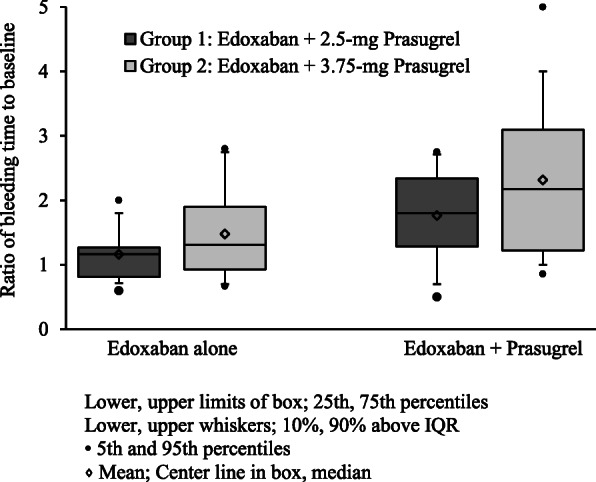
Changes in bleeding time induced by edoxaban administered alone and with prasugrel

The increase in BT is expressed as a GLSM of BT ratios (expressed as ratios to baseline) are shown in a box plot (Fig. 1). After 3 days of a once-daily dosing of 30 mg edoxaban, the GLSMs of BT ratios were 1.097 (90% CI 0.911–1.321) in Group 1 and 1.327 (90% CI 1.035–1.703) in Group 2. After coadministration of edoxaban and prasugrel for 5 days, BT was further prolonged. Especially in the edoxaban plus 3.75 mg prasugrel group, bleeding was continued over 10 min in 5 out of 12 subjects. The GLSMs of BT ratios were 1.581 (90% CI 1.197–2.087) in the edoxaban plus 2.5 mg prasugrel group and 1.996 (90% CI 1.482–2.690) in the edoxaban plus 3.75 mg prasugrel group. The GLSM increases in BT by prasugrel (edoxaban plus prasugrel/edoxaban alone) were 1.442 (90% CI 1.096–1.897) in the edoxaban plus 2.5 mg prasugrel group and 1.504 (90% CI 1.172–1.930) in the edoxaban plus 3.75 mg prasugrel group.

### Pharmacodynamic parameters of coagulation and platelet aggregation

**Fig. 2:**
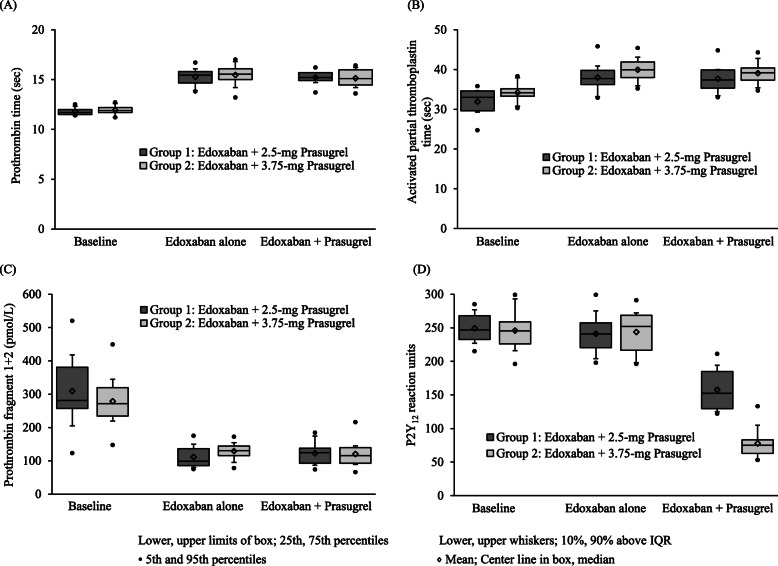
Changes in pharmacodynamic indices of coagulation and platelet aggregation induced by edoxaban administered alone and with prasugrel (**a**) Prothrombin time; (**b**) Activated partial thromboplastin time; (**c**) Prothrombin fragment 1 + 2; (**d**) P2Y_12_ reaction units

Figure 2 provides an overview of changes in pharmacodynamic parameters of coagulation and platelet aggregation. The administration of 30 mg edoxaban prolonged PT, in sec, from a mean of 11.8 at baseline to 15.3 (Group 1) and 11.9 at baseline to 15.5 (Group 2). With the addition of 2.5 mg or 3.75 mg prasugrel, the PT values were essentially unchanged for both groups (mean of 15.2 and 15.1). The aPTT, in sec, was also prolonged by edoxaban, from 31.9 at baseline to 38.0 (Group 1) and 34.2 at baseline to 39.9 (Group 2). Again, when 2.5 mg or 3.75 mg prasugrel was added on to the administration, the effect of edoxaban on aPTT, in sec, was unchanged (mean of 37.7 and 39.1). Edoxaban decreased F1 + 2, in pmol/L, from 309 at baseline to 112 (Group 1) and 279 at baseline to 129 (Group 2). The results of F1 + 2, were essentially unchanged when 2.5 mg or 3.75 mg prasugrel was added on to the administration of edoxaban (mean of 123 and 120). In the platelet aggregation assay, edoxaban essentially had no effect on PRU. After 2.5 mg or 3.75 mg prasugrel was added on to edoxaban, the PRU was reduced from a mean of 249 at baseline to 158 and 246 at baseline to 78, respectively.

### Pharmacokinetics

**Fig. 3:**
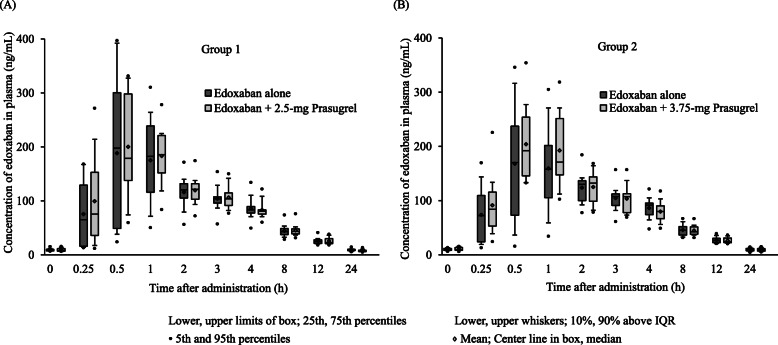
Effect of prasugrel on edoxaban concentrations in plasma (**a**) Group 1, administration of 30 mg edoxaban alone compared with 30 mg edoxaban plus 2.5 mg prasugrel; (**b**) Group 2, administration of 30 mg edoxaban alone compared with 30 mg edoxaban plus 3.75 mg prasugrel

The plasma edoxaban concentration profiles over 24 h were consistent, whether edoxaban was administered alone or with 2.5 mg or 3.75 mg prasugrel, as shown in Fig. 3. Also, prasugrel did not affect the PK parameters (C_max,ss_, AUC_tau,ss_, t_max,ss_) of edoxaban (Table 4).

**Table 4:** Pharmacokinetic parameters of edoxaban

	Group 1 30 mg edoxaban plus 2.5 mg prasugrel		Group 2 30 mg edoxaban plus 3.75 mg prasugrel	
	*n* = 12		*n* = 12	
	Edoxaban alone	Edoxaban plus prasugrel	Edoxaban alone	Edoxaban plus prasugrel
**C**_**max,ss**_**(ng/mL)**				
Mean (SD)	216.0 (112.4)	216.0 (80.4)	191.4 (79.7)	216.9 (68.1)
Median (IQR)	199.8 (127.6–300.5)	187.8 (169.0–298.3)	170.5 (134.5–237.3)	216.6 (157.3–269.3)
**AUC**_**tau,ss**_**(ng h/mL)**				
Mean (SD)	1059.4 (228.0)	1068.6 (189.8)	1070.4 (239.7)	1088.5 (223.2)
Median (IQR)	1025.0 (924.2–1152.4)	1005.8 (912.2–1216.7)	992.5 (953.1–1206.7)	1049.6 (930.4–1240.1)
**t**_**max,ss**_**(h)**				
Mean (SD)	1.2 (0.96)	0.92 (0.70)	1.0 (0.84)	0.71 (0.26)
Median (IQR)	0.75 (0.50–1.5)	0.75 (0.50–1.0)	0.50 (0.50–1.5)	0.50 (0.50–1.0)

*C*_*max,ss*_ steady state maximum plasma concentration, *AUC*_*tau,ss*_ steady state area under the plasma concentration-time curve, *t*_*max,ss*_ steady state time to reach maximum plasma concentration, *SD* standard deviation, *IQR* interquartile range

### Safety

Of the 24 participants, 16 had at least one occurrence of a treatment-emergent adverse event (AE) (16 positive results in the fecal occult blood test without stool blackening; 2, constipation). We observed 26 treatment-emergent AEs: 23 were reported as a treatment-related AE (positive result in the fecal occult blood test), and 3 were an AE unrelated to treatment (constipation) and most likely due to environmental changes at the hospital. We observed four positive results in the fecal occult blood test for edoxaban alone, 10 for edoxaban plus 2.5 mg prasugrel, and 9 for edoxaban plus 3.75 mg prasugrel and confirmed the recovery of all participants during the follow-up period. No serious adverse event was observed in the study. There were also no withdrawals or discontinuation of the study drug due to AEs or any other reason.

## Discussion

This study provided additional, preliminary safety results that concur with the 2018 joint European consensus document [2] recommending dual-therapy use of a DOAC plus a P2Y_12_ receptor inhibitor and also found no effect of prasugrel on PK and PD of edoxaban in healthy Japanese male subjects of normal weight (50 to 60 kg; BMI 21.1 ± 1.6 kg/m^2^) and advanced age (65 to 80 years).

Results of BT measured by the Ivy method were predictable and confirmatory for coadministration of edoxaban with prasugrel. Edoxaban (30 mg) alone prolonged BT 1.10- or 1.33-fold from baseline, and the addition of 2.5 mg and 3.75 mg prasugrel to edoxaban further prolonged BT 1.58- or 2.00-fold, respectively. The effect of prasugrel on BT was dose-dependent. This two-fold increase was comparable to the coadministration of 60 mg edoxaban with aspirin or naproxen, antiplatelet agents used in the study of Mendell et al. [13] and less than a 3.8-fold increase after the coadministration of 15 mg rivaroxaban with standard doses of clopidogrel [14]. The incidence of bleeding events caused by the concomitant treatment with 60 mg edoxaban plus aspirin has been evaluated in patients with peripheral artery disease who have undergone endovascular treatment [15]. The bleeding risk of 60 mg edoxaban plus aspirin was similar to that of standard dual antiplatelet therapy with clopidogrel plus aspirin [15]. Another clinical study showed that concomitant use of 15 mg rivaroxaban with clopidogrel in NVAF patients undergoing PCI was associated with a lower rate of clinically significant bleeding when compared to standard therapy with a vitamin K antagonist plus dual antiplatelet therapy [16]. Although BT may not always be correlated with bleeding risk in patients receiving antithrombotic agents [17, 18], the present data suggest that bleeding risk by dual-therapy edoxaban and prasugrel might be low.

The results of PD and PK parameters were also predictable and confirmatory. As expected, edoxaban alone prolonged PT and aPTT and decreased F1 + 2. Combined treatment with prasugrel had no additional effect on these parameters. In terms of platelet function, edoxaban alone had no effect on platelet aggregation, and only after administration of prasugrel, platelet aggregation was suppressed. Coadministration of prasugrel with edoxaban did not affect the PK parameters of edoxaban. These results of the combined administration of DOAC edoxaban and P2Y_12_ receptor inhibitor prasugrel are consistent with those reported previously for rivaroxaban and clopidogrel [14]; clopidogrel did not alter the anticoagulant effects and PK of rivaroxaban, whereas platelet aggregation was not affected by rivaroxaban.

This study reports safety results on the coadministration of edoxaban with prasugrel in a small study. All the treatment-emergent AEs were non-serious AEs. Most of the AEs were a positive fecal occult blood test, and all subjects with this AE did not require any treatment during the study period. Additionally, no study withdrawal or discontinuation due to AEs were observed.

It is also important to note that the reduced dose of edoxaban (30 mg) is used in 82% of elderly (≥75 years of age) AF patients in Japan [1]. Thus, for elderly AF patients undergoing PCI, clinicians can consider giving the reduced 30 mg dose of edoxaban with either the 2.5 mg or 3.75 mg dose of prasugrel. Since the effect of prasugrel on BT was dose-dependent, 2.5 mg prasugrel would be an option for patients whose body weight is ≤50 kg or who have other risk factors for bleeding.

There are several limitations of the study. First, this is a small clinical pharmacology study of short-term treatment in healthy elderly Japanese male subjects. Although we have set the duration of treatment for the purpose of reaching the steady states of PK variables for both edoxaban and prasugrel, according to the European consensus document [2] up to 12 months of treatment of dual therapy with an oral anticoagulant plus a P2Y_12_ receptor inhibitor are required for AF patients undergoing PCI. Large clinical studies are required to assess the concomitant use of edoxaban and prasugrel over a longer period in these patients, especially in patients who are at high-risk for bleeding. Second, it would be hard to predict bleeding risk of antithrombotic agents by BT results only [17, 18]. However, BT is currently the only method used to assess the risk of bleeding in clinical pharmacological studies of antithrombotic agents or combined effects of anticoagulant and antiplatelet agents [13, 14]. The increase in BT in a clinical study may indicate a potential for a higher risk of bleeding.

## Conclusions

Coadministration of the P2Y_12_ receptor inhibitor 2.5 mg and 3.75 mg prasugrel with the DOAC 30 mg edoxaban prolonged BT in healthy elderly Japanese male subjects. The BT prolongation was dependent on the dose of prasugrel. Prasugrel did not affect PD or PK parameters of edoxaban.

## Data Availability

The corresponding author can make available a minimal dataset upon reasonable request.

## Abbreviations

AE: Adverse event
aPTT: Activated partial thromboplastin time
BT: Bleeding time
CI: Confidence interval
DOAC: Direct oral anticoagulant
eGFR: Estimated glomerular filtration rate
F1 + 2: Prothrombin fragment 1 + 2
GLSM: Geometric least squares mean
NVAF: Nonvalvular atrial fibrillation
PCI: Percutaneous coronary intervention
PD: Pharmacodynamic
PK: Pharmacokinetic
PRU: P2Y_12_ reaction units
PT: Prothrombin time
SAS: Safety analysis set
